# Statement of Removal

**DOI:** 10.1080/15592324.2025.2604856

**Published:** 2025-12-21

This article has been removed in accordance with journal editorial policies.

Yang, D., Rao, R., Zheng, C., & Li, Y. (21 December 2025). GNA regulates rice spikelet formation via DEP1-enhanced repression of OsCKX2 and cytokinin modulation, *Plant Signaling & Behavior*, 21(1):2604856. https://doi.org/10.1080/15592324.2025.2604856

